# Interaction of obesity and proteins associated with the NLRP3 inflammasome following mild traumatic brain injury

**DOI:** 10.1038/s41598-024-61089-0

**Published:** 2024-05-03

**Authors:** Shawn R. Eagle, Mahesh K. Basantani, Jonathan Preszler, Natalie Sherry, Peyton McIntyre, Erin E. Kershaw, Ava M. Puccio, David O. Okonkwo

**Affiliations:** 1 Department of Neurological Surgery, University of Pittsburgh, 3550 Terrace St, Pittsburgh, PA 15261 USA; 2 Division of Endocrinology and Metabolism, Department of Medicine, University of Pittsburgh, Pittsburgh, PA USA; 3 Sanford Health, Bismarck, ND USA

**Keywords:** Diagnostic markers, Obesity

## Abstract

The NOD-like receptor pyrin domain-containing protein 3 (NLRP3) inflammasome has been associated with worse outcomes from severe traumatic brain injury (TBI). The NLRP3 inflammasome is also strongly associated with other pro-inflammatory conditions, such as obesity. Little is known about the potential effect of mild TBI (mTBI) on the NLRP3 inflammasome and the extent to which modifying factors, such as obesity, may augment the inflammatory response to mTBI. The purpose of this study was to evaluate the association of NLRP3 inflammasome proteins with obese body mass index (BMI ≥ 30) within 24 h of mTBI after presenting to a level 1 trauma center emergency department. This is a secondary analysis of prospectively enrolled patients with mTBI who presented to the emergency department of one U.S. Level 1 trauma center from 2013 to 2018 (n = 243). A series of regression models were built to evaluate the association of NLRP3 proteins obtained from blood plasma within 24 h of injury and BMI as well as the potential interaction effect of higher BMI with NLRP3 proteins (n = 243). A logistic regression model revealed a significant association between IL-18 (p < 0.001) in mTBI patients with obese BMI compared to mTBI patients with non-obese BMI (< 30). Moderation analyses revealed statistically significant interaction effects between apoptotic speck-like protein (ASC), caspase-1, IL-18, IL-1β and obese BMI which worsened symptom burden, quality of life, and physical function at 2 weeks and 6 months post-injury. Higher acute concentrations of IL-1β in the overall cohort predicted higher symptoms at 6-months and worse physical function at 2-weeks and 6-months. Higher acute concentrations of IL-18 in the overall cohort predicted worse physical function at 6-months. In this single center mTBI cohort, obese BMI interacted with higher acute concentrations of NLRP3 inflammasome proteins and worsened short- and long-term clinical outcomes.

## Introduction

Traumatic brain injury (TBI) remains a significant public health problem in the United States. Neuroinflammation is thought to be a key mechanism contributing to TBI impairments and prolonged clinical issues secondary to TBI^1,2^. An emerging body of research suggests that “inflammasomes” are a critical component of the neuroinflammatory response to injury. These multi-protein complexes are activated following trauma, resulting in release of caspase-1, interleukin-1 beta (IL-1β) and interleukin-18 (IL-18) into the blood. These inflammatory cytokines are associated with pyroptosis (i.e., inflammation-related neuronal cell death) and worse TBI outcomes^3,4^. IL-1β and IL-18, specifically, are considered end-points of the most-commonly studied inflammasome, NOD-like receptor pyrin domain-containing protein 3 (NLRP3).

Within the context of TBI, the NLRP3 mechanism has been almost exclusively investigated in animal models or human clinical studies of severe TBI. Following severe TBI, NLRP3 genes are upregulated by 3 h post-injury in blood. NLRP3, ASC, and caspase-1 mRNA are upregulated 6 h after severe TBI, while NLRP3 and caspase-1 mRNA continue to increase and peak by 7 days^5^. Protein expression for pro-caspase-1 and pro-IL-1β gradually increases up to 7 days^5^. Expression of these proteins releases caspase-1, IL-1β and IL-18 which lead to pyroptosis (i.e., inflammation-related neuronal cell death) and worse TBI outcomes^3,4^. IL-1β and IL-18 activate additional pro-inflammatory cytokines (i.e., IL-6, CRP), creating a positive feedback loop of inflammatory processes^4^. One recent study^6^ evaluated the serum levels of IL-1β and IL-18 at 2, 6, and 13 days after mild TBI (mTBI) in a small cohort (n = 25) of Australian-rules football players stratified by biological sex. The authors found no differences in concentrations of these cytokines at any timepoint^6^.

**Figure 1:**
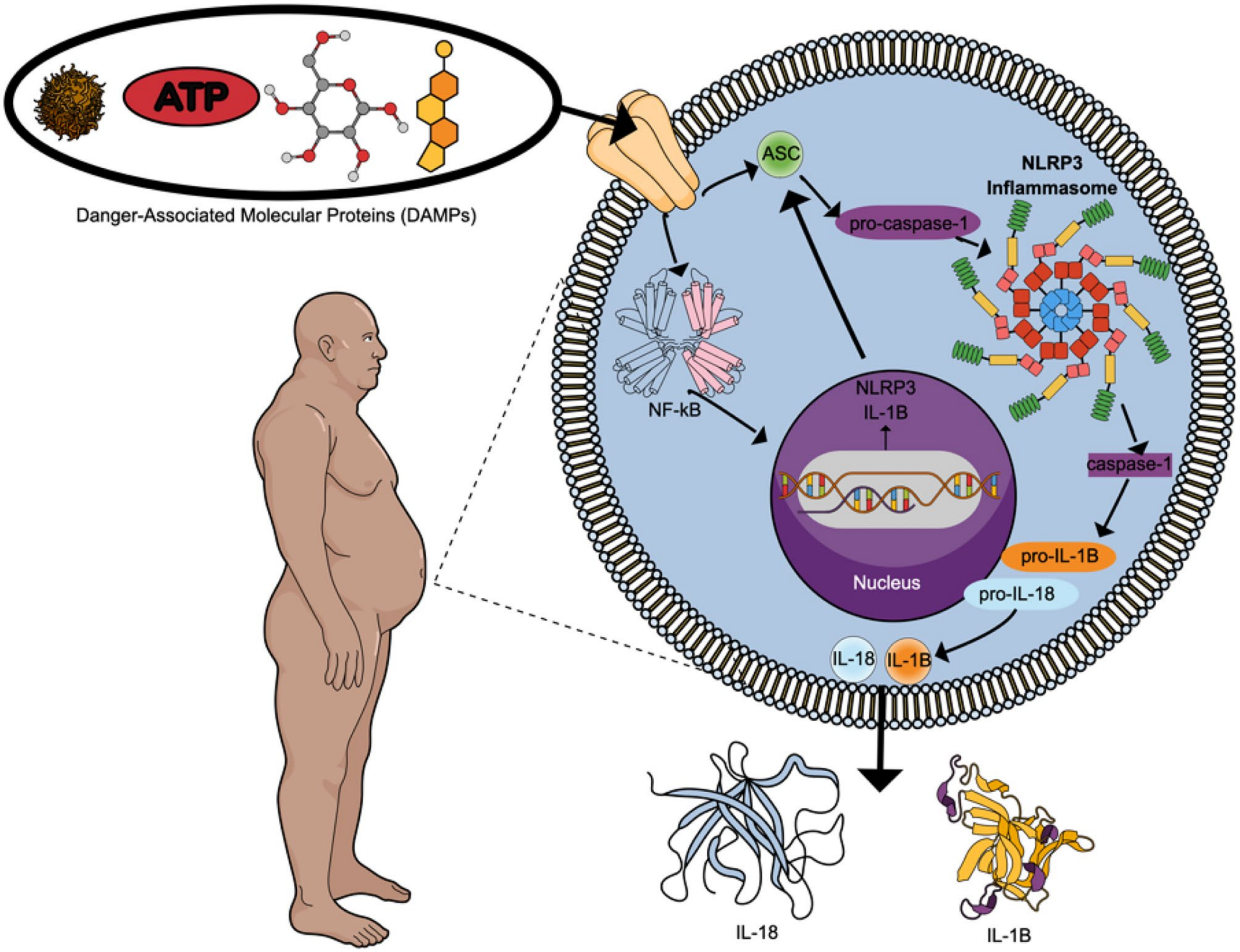
Theoretical depiction of pre-activation of the NLRP3 inflammasome in an obese person due to the increased presence of Danger Associated Molecular Proteins (DAMPs), such as amyloid beta, cholesterol and glucose^7,8,10^. Following severe TBI, NLRP3 genes are upregulated by 3 h post-injury. NLRP3, ASC, and caspase-1 mRNA are upregulated 6 h after severe TBI, while NLRP3 and caspase-1 mRNA continue to increase and peak by 7 days^5^. Protein expression for pro-caspase-1 and pro-IL-1β gradually increases up to 7 days^5^. Expression of these proteins releases caspase-1, IL-1β and IL-18 which lead to pyroptosis (i.e., inflammation-related neuronal cell death) and worse TBI outcomes^3,4^. IL-1β and IL-18 activate additional pro-inflammatory cytokines (i.e., IL-6, CRP), creating a positive feedback loop of inflammatory processes^4^.

The NLRP3 inflammasome is also strongly associated with other pro-inflammatory conditions, such as insulin resistance and obesity^7,8^. Prior studies have concluded that NLRP3 is activated by danger-associated molecular proteins (DAMPs) like higher blood glucose or cholesterol and actively contributes to obesity-induced inflammation and insulin resistance^8^. It is therefore possible that obesity may contribute to a more robust inflammatory response following physical trauma due to a pre-activation, or priming, of inflammasome architecture. However, few studies have investigated the role of obesity on mTBI recovery, despite multiple studies linking obesity to worse outcomes following severe TBI. One recent study found higher c-reactive protein the day after mTBI and at 2 weeks and 6 months post-injury in patients with obese body mass index (BMI) in comparison to those with normal BMI^9^. Obese patients also had higher IL-6 concentrations at 2 weeks and 6 months compared to normal patients. Higher IL-6 values have been associated with worse outcomes following mTBI. IL-6 can also activate the NLRP3 mechanism after TBI (see Fig. 1)^3,4^.

Given the high prevalence of both mTBI and obesity and their independent relationship to worse health outcomes and inflammation, investigating mechanisms linked to both conditions could yield important insights for treatment. The purpose of this study was to evaluate the association of NLRP3 inflammasome proteins with obese BMI within 24 h of mTBI. A secondary purpose of the study was to evaluate the potential moderating effect of these acute blood biomarkers on longitudinal symptoms and overall function within the first year after mTBI between participants with non-obese and obese BMI.

## Methods

### Study participants

This is a secondary analysis of prospectively enrolled patients with mTBI (Glasgow Coma Scale 13–15) who presented to the emergency department of one U.S. Level 1 trauma center from 2013 to 2018. Participants or their legally authorized representatives provided written informed consent to participate after being approached by a member of the research team in the hospital. Human subjects research approvals were obtained by the University of Pittsburgh institutional review board.

Participants were included in the study if they presented to the hospital within 24 h of external trauma to the head and met the American Congress of Rehabilitation Medicine’s definition of TBI^11^. The treating physician must also have deemed a head CT scan necessary for the patient to be enrolled. Exclusion criteria included pregnancy, incarceration, nonsurvivable physical trauma, debilitating mental health disorders, or neurological disease. Clinical outcome assessments were conducted at 2 weeks and 6 months post-injury.

### Clinical outcomes

The 16-item Rivermead Post-concussion Questionnaire (RPQ) measures severity of physical symptoms (e.g., headaches, dizziness, nausea) as well as cognitive, mood, and sleep disturbances associated with mTBI. Each item is rated on a Likert scale of 0 to 4, with 0 indicating the symptom was not experienced at all and 4 indicating the symptom was a severe problem within the past week, as compared with pre-injury status^12^. Because rating an item 1 is equivalent to “no more of a problem”, responses of 0 and 1 were merged into a category of 0. The maximum RPQ score is 64. The Quality of Life after Brain Injury—Overall Scale (QOLIBRI-OS) is a health-related quality-of-life instrument used for TBI patients with six items that comprise an overall score (range 0–100, lower scores indicate worse quality of life)^13^. The Short-Form 12 (SF-12) physical health is a summary scale using a subset of items from the SF-36 scales^14^. Summary scales are standardized to have higher scores indicate better health.

### Blood sample storage

Samples were processed and stored according to the Traumatic Brain Injury Common Data Elements Biospecimens and Biomarkers Working Group consensus recommendations for plasma and serum preparation^15^. Plasma and serum aliquots were prepared for each subject and frozen at − 80 °C for future analysis. All samples were deidentified using a unique study ID, specific to site and subject stored in the University of Pittsburgh Biospecimens Repository.

### Blood biomarker assays

Blood biomarkers were assayed using the commercially available traditional ELISA kits (Enzyme-Linked Immunosorbent Assay) or Meso Scale Discovery (MSD) biomarker assays according to manufacturer’s instructions.

### ASC ELISA

ASC was quantified using the Human PYCARD/ASC/TMS1 (Sandwich ELISA) ELISA Kit supplied by LS Bio (Catalog #: LS-F19843-1). The kit has a detection range between 0.156–10 ng/mL. The Detection Reagents A and B were diluted to prepare 1:100 Detection Reagent A and B Working Solutions. The lyophilized Standard Stock Solution (10 ng/mL) was diluted in 1 mL of Sample Diluent. The diluted Standard Stock Solution was used to prepare the series of Standards from 10 to 0.157 ng/mL. In a 96-well plate, 100 µL Standard, Blank and plasma samples were added. After 2 h incubation, with shaking, at 37 °C, the liquid was aspirated from each well, and 100 µL of Detection Reagent A Working Solution was added to each well. After 1 h incubation, with shaking, at 37 °C, the liquid was aspirated and the plate was washed 3 times with 350 µL of 1 × Wash Buffer. After the wash, 100 µL of Detection Reagent B Working Solution was added to each well. After 1 h incubation at 37 °C, the liquid was aspirated and the plate was washed 5 times with 350 µL of 1 × Wash Buffer. After the wash, 90 µL of TMB Substrate Solution was added to each well, and incubated, in dark, at 37 °C for 10–20 min. Then 50 µL of Stop Solution was added. The optical density was immediately determined at 450 nm in the microplate reader (Multiskan GO, Thermo Scientific).

### MSD biomarker assays

MSD assays are based on electrochemiluminescence as the detection method. IL-18, IL-1β and caspase-1 were assayed using MSD assay methods according to manufacturer’s instructions.

### IL-18 and caspase-1

IL-18 was quantified using the Human U-Plex IL-18 Assay and caspase-1 was quantified using the R-Plex Human Caspase-1 Antibody set (Catalog# B21K8-3). First, the capture antibody was coupled with the linker. 300 µL linker was mixed with 200 µL of biotinylated capture antibody by brief vortexing. After mixing, the linker-capture antibody solution was incubated at room temperature for 30 min. Finally, 200 µL of Stop Solution was added, and incubated at room temperature for 30 min. The linker-capture antibody solution was thus prepared. The linker-capture antibody complex was coated on sector plates at 50 µL solution per well. Next, the assay standards were prepared by adding 250 µL of diluent to the lyophilized calibrator. The diluted calibrator was used to prepare the series of standards. 25 µL of standards, blank and plasma samples were added in the linker-capture antibody complex coated sector plate. The plate was incubated, with shaking, at room temperature for 1 h. After 1 h, the plate was washed 3 times with 150 µL PBST (0.05% Tween 20 in 1 × PBS). After that, detection antibody solution was prepared by adding the diluent to the 100 × detection antibody. Then, 50 µL detection antibody solution was added to each well. The plate was incubated, with shaking, at room temperature for 1 h. After 1 h, the plate was washed 3 times with 150 µL PBST. Finally, 150 µL MSD Gold Read Buffer B was added to each well and the plate was read on the MSD instrument (MESO QuickPlex SQ 120MM).

### IL-1β

IL-1β was quantified using the S-Plex Human IL-1β Kit (Cat #K151ADSS-2). The Coating Solution was prepared immediately prior to use by combining Diluent 100, Biotin Human IL-1β antibody and S-Plex coating reagent C1. Then uncoated sector plate was washed 3 times with 150 µL PBST. The sector plates were coated by overnight incubation with 50 µL Coating Solution to each well. Next morning, Blocking Solution was prepared by combining Diluent 58 and 100 × Blocker S1. Next, the assay standards were prepared by adding 1000 µL of diluent to the lyophilized calibrator. The reconstituted calibrator solution was equilibrated at room temperature for 30 min. The diluted calibrator was used to prepare the series of standards. After that the overnight coated sector plate was washed 3 times with 150 µL PBST. After the wash, 25 µL of standards, blank and plasma samples were added, and the plate was incubated with shaking at room temperature for 1.5 h. During the incubation TURBO-BOOST Antibody Solution was prepared by combining Diluent 59 and TURBO-BOOST Human IL-1β Antibody. After the incubation, the plate was washed 3 times with 150 µL PBST. Then 50 µL TURBO-BOOST Antibody Solution was added to each well, and incubated with shaking at room temperature for 1 h. During the incubation Enhance Solution was prepared by combining MB (molecular biology) grade water, S-Plex Enhance E1, S-Plex Enhance E2 and S-Plex Enhance E3 reagents. After the incubation, the plate was washed 3 times with 150 µL PBST. Then 50 µL Enhance Solution was added to each well and incubated with shaking at room temperature for 30 min. During the incubation TURBO-TAG Detection Solution was prepared by combining MB grade water, S-Plex Detect D1 and S-Plex Detect D2 reagents. After the incubation, the plate was washed 3 times with 150 µL PBST. Then 50 µL TURBO-TAG Detection Solution was added to each well, and incubated with shaking at 27 °C for 1 h. After the incubation, the plate was washed 3 times with 150 µL PBST. Finally, 150 µL MSD Gold Read Buffer B was added to each well and the plate was read on the MSD instrument (MESO QuickPlex SQ 120MM). The data generated from MESO QuickPlex SQ 120MM instrument was imported into MSD Discovery Workbench software for further analysis.

### Statistical analysis

Independent samples t-tests and chi-square tests were used to compare groups on demographic variables. Due to violating normality assumptions, the biomarker values were transformed with a natural log function which was used for all analyses. A logistic regression model was built to evaluate the association of NLRP3 proteins and participants with obese BMI (≥ 30) compared to non-obese BMI (BMI < 30). An ordinal regression model was built to evaluate the association of NLRP3 proteins and participants with non-obese BMI (< 30), obese BMI (30–34.9) and severely obese BMI (≥ 35.0). To evaluate the potential moderating effect of NLRP3 proteins on clinical outcomes at two-weeks and six-months post mTBI, six linear regression models were built with RPQ total score, QOLIBRI-OS total score, and SF-12 physical score as the dependent variables at both timepoints. Included predictors in each linear regression model were ASC, caspase-1, IL-18, and IL-1β within 24 h of mTBI, obese BMI vs. non-obese BMI, and interaction terms for each biomarker and obese vs. non-obese BMI. Diabetes, hypertension and hyperlipidemia were included as covariates in every model. The above methods were repeated to evaluate the potential moderating association between NLRP3 proteins with non-obese BMI, obese BMI, and severely obese BMI and interaction terms on clinical outcomes at each timepoint. Conditional effects of the predictor based upon obesity group status are reported for significant interaction terms. Each model underwent a bootstrap procedure (5000 iterations) to increase accuracy of coefficients and 95% CI. Heteroscedasticity-consistent inference was applied to each moderation model to increase robustness of the standard errors and ensure meeting the heteroscedasticity assumption^16^. Post-hoc evaluations were conducted for each included variable with partial correlations and variance inflation factor calculations (VIF). Correlation strength < 0.8 and VIF < 4 were deemed acceptable cutoffs for meeting the multicollinearity assumption^17^. All models met these parameters. Because the present work was an exploratory analysis of a dataset for which the original study was not powered to detect differences, corrections for multiple comparisons were not applied. The PROCESS v4.2 package in IBM SPSS Statistics v29.0.1 was used for the moderation regression analyses^18^. Statistical significance was p < 0.05.

### Human ethics and consent to participate

Participants or their legally authorized representatives provided written informed consent to participate after being approached by a member of the research team in the hospital. Human subjects research approvals were obtained by the University of Pittsburgh institutional review board.

## Results

### Summary of overall cohort

Descriptive statistics for the overall sample can be viewed in Table 1 (n = 190). The overall cohort was 42.4 ± 17.5 years of age, 32.1% female (n = 61), 84.7% had a Glasgow Coma Scale of 15 at admission (n = 161), 84.7% white (n = 161) and 2.1% Hispanic/Latino ethnicity. Approximately one-third of the sample had a positive head computed tomography scan at admission (n = 52; 30.4%). Obese participants were more likely to have diabetes, hypertension, and hyperlipidemia compared to non-obese participants.

**Table 1:** Descriptive statistics for the overall sample.

	Obese BMI (n = 61)	Non-obese BMI (n = 129)	p
Age in years	45.2 ± 16.7	41.0 ± 17.8	0.13
Female sex	21 (34.4)	40 (31.0)	0.74
Diabetes	10 (16.4)	1 (0.8)	< 0.001*
Hypertension	20 (32.8)	17 (13.2)	< 0.001*
Hyperlipidemia	7 (11.5)	3 (2.3)	0.01*
Headache	0 (0.0)	1 (0.8)	1.00
Pulmonary disease	10 (16.4)	14 (10.9)	0.24
Renal disease	6 (10.0)	8 (6.2)	0.37
Anxiety	12 (19.7)	17 (13.2)	0.20
Depression	11 (18.0)	18 (14.0)	0.41
Sleep disorder	0 (0.0)	3 (2.3)	0.55
ASC (ng/mL)	1.00 ± 0.7	0.87 ± 0.4	0.17
Caspase-1 (pg/mL)	5185.35 ± 4114.3	6387.64 ± 5616.4	0.19
IL-18 (pg/mL)	876.24 ± 477.1	626.5 ± 295.0	0.002*
IL-1β (fg/mL)	203.33 ± 978.4	79.25 ± 127.6	0.44
RPQ at 2 weeks	23.0 ± 19.0	17.0 ± 14.2	0.03*
RPQ at 6 months	16.3 ± 17.7	13.4 ± 14.4	0.28
QOLIBRI-OS at 2 weeks	47.0 ± 26.1	53.8 ± 25.6	0.14
QOLIBRI-OS at 6 months	56.6 ± 28.2	63.1 ± 27.4	0.18
SF-12 physical at 2 weeks	32.1 ± 11.0	34.9 ± 11.7	0.18
SF-12 physical at 6 months	43.8 ± 11.7	46.8 ± 10.4	0.12

*ASC* apoptotic speck-like protein, *IL* interleukin, *RPQ* Rivermead Post-concussion Questionnaire, *QOLIBRI-OS* Quality of Life after Brain Injury Overall Scale, *SF-12* Short Form 12, *ng/mL* nanogram per milliliter, *pg/mL* picogram per milliliter, *fg/mL* femtogram per milliliter.

*Statistically significant at p ≤ 0.05.

### Association between NLRP3 markers and obesity

A logistic regression model revealed a significant association between IL-18 (β = 2.01; p < 0.001) and obese BMI compared to non-obese BMI. There was no statistically significant association between ASC (β = 0.28; p = 0.65), caspase-1 (β = 0.16; p = 0.57) and IL-1β (β = − 0.27; p = 0.27) in mTBI patients with obese BMI compared to mTBI patients with non-obese BMI. An ordinal regression model revealed an association between IL-18 (β = 2.11; p < 0.001) and obese BMI. ASC (β = 0.22; p = 0.71), caspase-1 (β = 0.24; p = 0.35) and IL-1β (β = − 0.35; p = 0.15) did not have a statistically significant association with mTBI patients with obese and severely obese BMI compared to patients with non-obese BMI.

### Moderation analyses of NLRP3 markers and obesity on clinical outcomes at 2 weeks and 6 months after mTBI

Model predictors for each outcome at 2 weeks and 6 months can be viewed in Table 2. The logistic regression model to predict RPQ total score at 2 weeks had an R^2^ = 0.18 and included the interaction term between obese BMI and ASC (p = 0.01) as a significant predictor, such that higher ASC increased RPQ symptom scores at 2-weeks in the obese group (conditional effect: 23.6; p = 0.01). The model to predict RPQ score at 6-months had an R^2^ = 0.18 and included IL-1β (p = 0.02) and the interaction of IL-1β with obese BMI (p = 0.02) as significant predictors, such that higher IL-1β was associated with higher RPQ symptom scores at 6-months overall and higher IL-1β increased symptom scores in the non-obese group at 6-months (conditional effect = 4.9; p = 0.02).

**Table 2:** Linear regression to predict outcomes at 2 weeks and 6 months post-mild traumatic brain injury.

	Coefficient	95% CI	p
RPQ 2 weeks			
Obese BMI (yes/no)	− 30.7	− 260.9, 199.5	0.79
ASC	− 3.9	− 14.7, 6.8	0.47
Caspase-1	− 0.1	− 5.2, 5.0	0.97
IL-18	4.9	− 6.0, 15.8	0.38
IL-1β	1.4	− 3.7, 6.5	0.59
Obese BMI × ASC	27.5	5.9, 49.1	0.01*
obese BMI × caspase-1	9.2	− 3.3, 21.6	0.15
Obese BMI × IL-18	− 4.6	− 28.7, 19.6	0.71
Obese BMI × IL-1β	− 0.6	− 8.2, 7.0	0.88
Hypertension	− 1.2	− 10.5, 8.0	0.79
Hyperlipidemia	− 0.5	− 17.2, 16.1	0.95
Diabetes	− 2.7	− 19.3, 13.9	0.75
RPQ 6 months			
Obese BMI (yes/no)	30.4	− 161.3, 222.1	0.75
ASC	3.8	− 7.6, 15.3	0.51
Caspase-1	− 0.3	− 4.8, 4.2	0.90
IL-18	6.6	− 3.8, 17.0	0.21
IL-1β	4.9	0.9, 8.9	0.02*
Obese BMI × ASC	4.9	− 18.0, 27.7	0.67
Obese BMI × caspase-1	7.1	− 2.8, 16.9	0.16
Obese BMI × IL-18	− 7.1	− 27.6, 13.4	0.49
Obese BMI × IL-1β	− 10.2	− 18.8, − 1.5	0.02*
Hypertension	0.1	− 8.6, 8.8	0.98
Hyperlipidemia	− 9.3	− 23.0, 4.4	0.18
Diabetes	11.8	− 4.5, 28.1	0.15
QOLIBRI-OS 2 weeks			
Obese BMI (yes/no)	33.2	− 247.9, 314.3	0.81
ASC	1.1	− 17.3, 199.4	0.91
Caspase-1	− 1.8	− 8.7, 5.2	0.62
IL-18	− 3.4	− 15.7, 8.8	0.58
IL-1β	− 0.7	− 5.6, 4.3	0.79
Obese BMI × ASC	− 44.5	− 78.5, − 10.6	0.01*
Obese BMI × caspase-1	− 8.5	− 24.0, 7.1	0.28
Obese BMI × IL-18	− 2.5	− 27.5, 32.4	0.87
Obese BMI × IL-1β	2.1	− 6.8, 11.1	0.64
Hypertension	7.3	− 7.4, 22.0	0.32
Hyperlipidemia	9.0	− 25.8, 43.7	0.61
Diabetes	− 18.3	− 41.6, 5.1	0.12
QOLIBRI-OS 6 months			
Obese BMI (yes/no)	− 205.2	− 512.0, 101.6	0.19
ASC	7.7	− 13.9, 29.2	0.48
Caspase-1	− 1.9	− 10.0, 6.2	0.64
IL-18	− 5.1	− 22.8, 12.6	0.57
IL-1β	− 8.1	− 17.2, 1.1	0.08
Obese BMI × ASC	− 26.8	− 66.1, 12.6	0.18
Obese BMI × caspase-1	− 1.6	− 17.8, 14.5	0.84
Obese BMI × IL-18	20.1	− 13.7, 53.9	0.24
Obese BMI × IL-1β	19.7	4.6, 34.8	0.05
Hypertension	− 16.1	− 33.1, 1.0	0.06
Hyperlipidemia	30.8	1.2, 60.4	0.04*
Diabetes	− 20.7	− 47.6, 6.3	0.13
SF-12 physical at 2 weeks			
Obese BMI (yes/no)	17.2	− 166.5, 150.8	0.80
ASC	− 3.8	− 11.5, 3.9	0.32
Caspase-1	0.5	− 3.2, 4.2	0.79
IL-18	0.1	− 9.1, 9.4	0.98
IL-1β	− 3.0	− 5.4, − 0.7	0.01*
Obese BMI × ASC	− 11.2	− 24.3, 1.9	0.09
Obese BMI × caspase-1	− 2.4	− 8.9, 4.1	0.47
Obese BMI × IL-18	− 0.9	− 15.3, 13.5	0.90
Obese BMI × IL-1β	1.1	− 1.9, 4.1	0.46
Hypertension	− 0.5	− 5.9, 4.9	0.85
Hyperlipidemia	− 0.1	− 7.7, 7.5	0.98
Diabetes	− 4.7	− 8.8, − 0.6	0.02*
SF-12 physical at 6 months			
Obese BMI (yes/no)	− 106.9	− 205.3, − 8.5	0.03*
ASC	− 2.1	− 10.1, 6.0	0.61
Caspase-1	− 2.2	− 5.0, 0.7	0.14
IL-18	− 5.0	− 9.9, − 0.1	0.05
IL-1β	− 4.6	− 7.2, − 2.1	0.00*
Obese BMI × ASC	− 12.6	− 26.1, 0.8	0.07
Obese BMI × caspase-1	1.4	− 3.5, 6.2	0.57
Obese BMI × IL-18	11.0	− 0.7, 22.7	0.06
Obese BMI × IL-1β	5.8	0.2, 11.4	0.04*
Hypertension	− 4.6	− 11.0, 1.9	0.17
Hyperlipidemia	7.5	− 7.1, 22.1	0.31
Diabetes	− 16.0	− 29.0, − 3.1	0.02*

*ASC* apoptotic speck-like protein, *IL* interleukin, *RPQ* Rivermead Post-concussion Questionnaire, *QOLIBRI-OS* Quality of Life after Brain Injury Overall Scale, *SF-12* Short Form 12, *ng/mL* nanogram per milliliter, *pg/mL* picogram per milliliter, *fg/mL* femtogram per milliliter.

*Statistically significant at p < 0.05.

The model to predict QOLIBRI-OS at 2-weeks had an R^2^ = 0.18 and included the interaction of obese BMI and ASC as a significant predictor (p = 0.01), such that higher ASC reduced quality of life scores in the obese group (conditional effect in the obese group: − 43.5, < 0.001). The model to predict QOLIBRI-OS at 6-months had an R^2^ = 0.21 and included hyperlipidemia as a significant predictor.

The model to predict SF-12 physical at 2-weeks had an R^2^ = 0.20 and included IL-1β (p = 0.01) and diabetes (p = 0.02) as significant predictors, such that higher IL-1β and reporting comorbid diabetes worsened SF-12 physical score at 2-weeks. The model to predict SF-12 physical at 6-months had an R^2^ = 0.31 and included IL-1β as a significant predictor (p = 0.00), IL-18 (p = 0.05), the interaction of obese BMI and IL-1β (p = 0.04), and diabetes (p = 0.02), and reporting comorbid diabetes worsened SF-12 physical score at 6-months. The interaction of obese BMI and IL-1β had a conditional effect of -4.6 (p < 0.001) for the non-obese group, such that higher IL-1β worsened SF-12 physical scores at 6-months for the non-obese group only.

### Moderation analyses of NLRP3 markers and severe obesity on clinical outcomes at 2 weeks and 6 months after mTBI

Model predictors for each outcome at 2 weeks and 6 months can be viewed in Table 3. The logistic regression model to predict RPQ at 2-weeks had an R^2^ = 0.32 and included obese BMI (p = 0.01), severely obese BMI (p = 0.01), the interaction of severely obese BMI and ASC (conditional effect: 29.0, p < 0.001), interaction of obese BMI with caspase-1 (conditional effect: 18.8, p < 0.001), interaction of severely obese BMI and IL-18 (p = 0.01), and diabetes (p = 0.04) as significant predictors. The conditional effect of higher IL-18 (23.2, p = 0.02) was observed for the obese group only. The model to predict RPQ at 6-months had an R^2^ = 0.27 and included IL-1β (p = 0.03), the interaction of obese BMI and caspase-1 (conditional effect: 15.2, p < 0.001) and the interaction of severely obese BMI and IL-1β (p = 0.02) as significant predictors, such that higher IL-1β and higher caspase-1 in the obese group were associated with higher symptoms at 6-months. The conditional interaction effect with IL-1β was observed for the non-obese group only (4.5, p = 0.03).

**Table 3:** Linear regression to predict outcomes at 2 weeks and 6 months post-mild traumatic brain injury.

	Coefficient	95% CI	p
RPQ 2 weeks			
Obese BMI (REF: non-obese BMI)	− 275.4	− 475.0, − 75.6	0.01*
Severely obese BMI (REF: non-obese BMI)	270.7	66.9, 474.5	0.01*
ASC	− 3.6	− 14.2, 7.1	0.51
Caspase-1	− 0.1	− 5.2, 5.1	0.98
IL-18	5.0	− 5.9, 15.8	0.36
IL-1β	1.1	− 4.0, 6.2	0.66
Obese BMI × ASC	15.3	− 16.5, 47.2	0.34
Severely obese BMI × ASC	32.5	16.3, 48.7	0.00*
Obese BMI × caspase-1	18.8	8.0, 29.6	0.00*
Severely Obese BMI × caspase-1	− 6.9	− 23.5, 9.7	0.41
Obese BMI × IL-18	18.3	− 4.5, 41.0	0.11
Severely obese BMI × IL-18	− 27.5	− 48.6, − 6.5	0.01*
Obese BMI × IL-1β	1.9	− 4.7, 8.5	0.57
Severely obese BMI × IL-1β	− 2.8	− 16.0, 10.4	0.68
Hypertension	− 2.6	− 12.6, 7.5	0.61
Hyperlipidemia	3.0	− 10.0, 15.8	0.65
Diabetes	− 10.0	− 19.4, − 0.7	0.04*
RPQ 6 months			
Obese BMI (REF: non-obese BMI)	− 129.0	− 353.6, 95.7	0.26
Severely obese BMI (REF: non-obese BMI)	43.6	− 209.6, 296.8	0.73
ASC	3.8	− 7.8, 15.5	0.51
Caspase-1	0.0	− 4.4, 4.4	0.99
IL-18	6.1	− 4.4, 16.6	0.25
IL-1β	4.5	0.6, 8.5	0.03*
Obese BMI × ASC	10.2	− 21.6, 41.9	0.53
Severely obese BMI × ASC	− 21.9	− 51.0, 7.1	0.14
Obese BMI × caspase-1	15.2	4.8, 25.5	0.00*
Severely obese BMI × caspase-1	− 3.8	− 18.2, 10.5	0.59
Obese BMI × IL-18	3.3	− 19.7, 26.2	0.78
Severely obese BMI × IL-18	9.0	− 18.0, 36.0	0.51
Obese BMI × IL-1β	− 3.7	− 11.9, 4.6	0.38
Severely obese BMI × IL-1β	− 20.0	− 36.2, − 3.9	0.02*
Hypertension	1.9	− 7.6, 11.3	0.69
Hyperlipidemia	− 9.3	− 19.4, 0.9	0.07
Diabetes	1.6	− 11.4, 14.6	0.81
QOLIBRI-OS 2 weeks			
Obese BMI (REF: non-obese BMI)	240.4	− 14.4, 495.2	0.06
Severely obese BMI (REF: non-obese BMI)	− 263.8	− 771.7, 244.1	0.30
ASC	0.7	− 17.6, 18.9	0.94
Caspase-1	− 1.7	− 8.7, 5.3	0.63
IL-18	− 3.7	− 15.8, 8.5	0.55
IL-1β	− 0.4	− 5.4, 4.5	0.86
Obese BMI × ASC	− 20.3	− 66.7, 26.2	0.39
Severely obese BMI × ASC	− 41.8	− 76.1, − 7.4	0.02*
Obese BMI × caspase-1	− 17.6	− 31.4, − 3.7	0.01*
Severely obese BMI × caspase-1	17.6	− 18.1, 53.3	0.33
Obese BMI × IL-18	− 13.8	− 43.2, 15.6	0.35
Severely obese BMI × IL-18	5.5	− 28.1, 39.0	0.75
Obese BMI × IL-1β	− 3.0	− 10.0, 4.1	0.40
Severely obese BMI × IL-1β	18.9	0.5, 37.3	0.05
Hypertension	9.3	− 6.7, 25.3	0.25
Hyperlipidemia	6.5	− 18.3, 31.2	0.60
Diabetes	− 11.9	− 30.1, 6.3	0.20
QOLIBRI-OS 6 months			
Obese BMI (REF: non-obese BMI)	82.0	− 246.9, 410.8	0.62
Severely obese BMI (REF: non-obese BMI)	− 224.9	− 589.4, 139.6	0.22
ASC	8.0	− 13.9, 29.9	0.47
Caspase-1	− 2.5	− 10.3, 5.3	0.52
IL-18	− 3.9	− 21.8, 14.0	0.67
IL-1β	− 7.5	− 16.8, 1.8	0.11
Obese BMI × ASC	− 31.8	− 83.4, 19.8	0.22
Severely obese BMI × ASC	5.6	− 40.8, 52.0	0.81
Obese BMI × caspase-1	− 14.1	− 32.8, 4.7	0.14
Severely obese BMI × caspase-1	10.8	− 12.4, 33.9	0.36
Obese BMI × IL-18	− 0.3	− 36.0, 35.3	0.98
Severely obese BMI × IL-18	− 2.6	− 46.3, 41.1	0.90
Obese BMI × IL-1β	6.4	− 9.4, 22.1	0.42
Severely obese BMI × IL-1β	41.3	15.0, 67.6	0.00*
Hypertension	− 21.1	− 40.0, − 2.5	0.03*
Hyperlipidemia	31.7	7.9, 55.4	0.01*
Diabetes	− 6.1	− 29.7, 17.5	0.61
SF-12 Physical 2 weeks			
Obese BMI (REF: non-obese BMI)	19.5	− 130.6, 169.6	0.80
Severely obese BMI (REF: non-obese BMI)	− 32.9	− 204.0, 138.3	0.70
ASC	− 3.8	− 11.5, 3.9	0.33
Caspase-1	0.5	− 3.2, 4.2	0.79
IL-18	0.1	− 9.1, 9.4	0.98
IL-1β	− 3.0	− 5.4, − 0.7	0.01*
Obese BMI × ASC	− 5.7	− 21.0, 9.6	0.46
Severely obese BMI × ASC	− 6.2	− 19.0, 6.7	0.34
Obese BMI × caspase-1	− 2.6	− 10.0, 4.8	0.49
Severely obese BMI × caspase-1	5.0	− 6.8, 16.9	0.40
Obese BMI × IL-18	0.1	− 15.9, 16.2	0.99
Severely obese BMI × IL-18	− 5.9	− 18.9, 7.2	0.37
Obese BMI × IL-1β	− 0.4	− 3.9, 3.1	0.82
Severely obese BMI × IL-1β	7.2	− 1.6, 15.9	0.11
Hypertension	− 0.5	− 6.4, 5.4	0.85
Hyperlipidemia	− 1.5	− 10.4, 7.4	0.73
Diabetes	− 5.5	− 10.3, − 0.8	0.02*
SF-12 physical 6 months			
Obese BMI (REF: non-obese BMI)	− 35.6	− 201.7, 130.3	0.67
Severely obese BMI (REF: non-obese BMI)	− 140.2	− 232.9, − 47.6	0.00*
ASC	− 2.1	− 10.2, 6.0	0.60
Caspase-1	− 2.2	− 5.1, 0.6	0.13
IL-18	− 4.9	− 9.7, − 0.2	0.04*
IL-1β	− 4.5	− 7.1, − 1.9	0.00*
Obese BMI × ASC	− 15.9	− 35.6, 2.8	0.09
Severely obese BMI × ASC	− 4.2	− 22.1, 13.6	0.64
Obese BMI × caspase-1	− 2.3	− 10.7, 6.1	0.59
Severely obese BMI × caspase-1	6.0	− 0.5, 12.5	0.07
Obese BMI × IL-18	5.6	− 11.1, 22.3	0.51
Severely obese BMI × IL-18	10.5	− 4.0, 25.1	0.15
Obese BMI × IL-1β	4.6	− 3.5, 12.7	0.26
Severely obese BMI × IL-1β	4.8	− 3.0, 12.6	0.22
Hypertension	− 4.8	− 12.1, 2.6	0.20
Hyperlipidemia	7.5	− 4.7, 19.7	0.22
Diabetes	− 12.8	− 26.6, 1.1	0.07

*ASC* apoptotic speck-like protein, *IL* interleukin, *RPQ* Rivermead Post-concussion Questionnaire, *QOLIBRI-OS* Quality of Life after Brain Injury Overall Scale, *SF-12* Short Form 12, *ng/mL* nanogram per milliliter, *pg/mL* picogram per milliliter, *fg/mL* femtogram per milliliter.

*Statistically significant at p < 0.05.

The model to predict QOLIBRI-OS at 2-weeks had an R^2^ = 0.27 and included the interaction of severely obese BMI with ASC (conditional effect: − 41.1, p = 0.02), and the interaction of severely obese BMI with caspase-1 (conditional effect: − 19.3, p < 0.001) as significant predictors, such that higher ASC and caspase-1 in the severely obese BMI group were associated with lower QOLIBRI-OS scores at 2-weeks. The logistic regression model to predict QOLIBRI-OS at 6-months had an R^2^ = 0.28 and included hypertension, hyperlipidemia, and the interaction term of severely obese BMI and IL-1β (p < 0.001) as a significant predictor, such that reporting hypertension, hyperlipidemia or having higher IL-1β in the severely obese group reduced QOLIBRI-OS scores at 6-months.

The model to predict SF-12 physical at 2-weeks had an R^2^ = 0.23 and included IL-1β (p = 0.01) and diabetes (p = 0.02) as significant predictors, such that higher IL-1β and reporting diabetes reduced SF-12 physical scores at 2-weeks. The model to predict SF-12 physical at 6-months had an R^2^ = 0.34 and included severely obese BMI (p < 0.001), IL-18 (p = 0.04), IL-1β (p < 0.001) as significant predictors, such that severely obese BMI and higher IL-18 and IL-1β reduced SF-12 physical scores at 6-months.

## Discussion

In this single-center study, we evaluated the relationship between acute ASC, caspase-1, IL-18 and IL-1β with BMI following mTBI. Obesity interacted with inflammatory proteins to worsen short- and long-term clinical outcomes after mTBI. Proteins associated with the NLRP3 inflammasome (i.e., ASC, caspase-1, IL-18 and IL-1β) each interacted with BMI groups to worsen at least one of the clinical outcomes at either 2 weeks or 6 months post-injury. These results suggest that higher BMI may augment the acute cytokine response to mTBI and, specifically, NLRP3 inflammasome activation in the mTBI patient may be predictive of clinical outcomes. The results of this study support deeper investigation into the NLRP3 inflammasome as a potential mechanism of this relationship (Fig. 1).

IL-18 and IL-1β are the key cytokine endpoints of the NLRP3 inflammasome. In the present study, IL-18 was the only NLRP3 protein studied which was associated with obesity status, but it was only predictive of SF-12 physical scores at 6-months and RPQ scores at 2-weeks in the severe obesity model (Table 3). Conversely, IL-1β (either alone or as an interaction with BMI groups) was significantly associated with the outcome in 7 of 12 possible regression models with a statistical trend in another 2 models (p = 0.05). Prior work has shown that IL-18 levels can be elevated in obese participants, as the cytokine is associated with maintaining energy homeostasis^19,20^. IL-18 may therefore be a sign of chronic inflammation in obese patients with less predictive ability following mTBI compared to IL-1β. Total and regional body fat percentage was not available for the present analysis but may have resulted in stronger associations between the blood markers than BMI and potentially influenced worse outcomes. Future work needs to evaluate the association of body fat with NLRP3 activation after mTBI and its association with mTBI outcomes.

While it is well-known that higher acute inflammatory response is associated with worse mTBI outcomes, there is limited data available about whether IL-18 and IL-1β specifically are associated with worse outcomes after mTBI in humans^6^. Using a multivariate proteomic panel, Huie et al.^1^ reported on a principal components analysis which identified a pro-inflammatory panel associated with mTBI outcomes. The final principal component grouping did not include IL-18 or IL-1β. In another study, IL-1β was able to discriminate mTBI from controls but did not have prognostic utility for short or long-term outcomes^2^. Many studies have been unable to detect circulating IL-1β in populations with concussion^21,22^, leading authors to conclude that perhaps that mechanism of injury is not sufficient to elicit a pathophysiological response which includes release of IL-18 and IL-1β. Prior work has possibly been limited by sampling blood too late post-injury, as both IL-18 and IL-1β have short half-lives (i.e., approximately 4 h) in comparison to other mTBI markers^6^. The results of this study suggest that, when sampled within the first day of injury, IL-1β can have predictive utility in an mTBI population who presents to a hospital emergency department for evaluation.

Blood samples were only available for this study within 24 h of injury. In the context of patients with severe TBI, activation of mRNA and subsequent protein expression for caspase-1, IL-18 and IL-1β peak around 7 days post-injury^23^. Very little is known about the stages of activation and possible perpetuation of the NLRP3 inflammasome following mTBI. It is possible that the evolution of NLRP3 activation after mTBI (beyond 24 h post-injury) could influence clinical outcomes at later timepoints. Modifying factors, such as comorbid obesity, diabetes, or cardiovascular disease, may contribute to continuous upregulation of NLRP3 following head trauma and contribute to the pathogenesis of chronic mTBI symptoms or impairments^4,8,24,25^. Future research is necessary to characterize the temporal activation of NLRP3 after mTBI and whether those temporal characteristics influence long-term outcomes.

Research has recently begun to investigate the impact of NLRP3-inhibitors on recovery from TBI in animal models. Ismael et al.^26^ reported a significant improvement in neurological function and reduced cerebral edema following administration of an NLRP3 inhibitor (i.e., MCC950) at 1 and 3 h post-TBI in a controlled cortical impact mouse model. Treatment with MCC950 inhibited inflammasome priming by downregulating the production of nuclear factor kappa B and caspase-1^26^. Caspase-1 and IL-1β production were also repressed in treated mice^26^. Similarly, in a rat model of repeated low-level blast exposure, treatment with MCC950 reduced NLRP3 expression and IL-1β release while improving short-term memory deficits^27^. Utilizing MCC950 has also been of interest to mitigate NLRP3-mediated inflammation related to neurodegenerative disease in animal models. Naeem et al.^28^ recently reported that MCC950 could be a viable treatment for Alzheimer’s Disease (AD), by demonstrating the drug’s ability to reverse amyloid beta plaque formation and improve cognitive function in rats with AD. No trials to date have used MCC950 to treatment mTBI or patients with chronic mTBI symptoms, but future work should consider the potential role of NLRP3 inhibitors in this population.

### Limitations

These findings should be interpreted with some limitations. The results presented here should be considered preliminary as they represent participants from a single center with limited sample size. Due to the exploratory nature of the study, corrections were for multiple comparisons were not applied which could increase risk for Type I error. The regression models accounted for 18–34% of the variance in a given clinical outcome at a specific timepoint, indicating other variables influence outcome prediction beyond NLRP3 inflammatory markers and obese BMI. Diabetes, hypertension, and hyperlipidemia were included as covariates in all moderation regression models due to higher proportions of obese participants reporting these conditions. Obesity is highly associated with comorbidities which can increase inflammation, which was not explored extensively due to limited sample size. Future work should evaluate differences in NLRP3 inflammasome proteins after mTBI based upon clusters of obesity and obesity-related medical conditions. High BMI can be biased by factors such as more muscle mass, which could result in less variance accounted for between BMI and the blood markers. Also, clinical outcome in this study was measured by self-reported symptoms and future studies should include objective measures of clinical outcome and functional status. Blood was collected for all participants within 24 h of injury, but the precise time from injury until blood collection is not known. Polytrauma data were not available for the present study and could have impacted inflammatory response. These results are only generalizable to mTBI patients seen at a level 1 trauma center emergency department.

## Conclusion

In this single-center study of participants with mTBI seen at a level 1 trauma center emergency department, acute levels of ASC, caspase-1, IL-18, and IL-1β interacted with obese BMI and were significant predictors of worse short (2 weeks) and long-term (6 months) outcomes. Moderation analyses revealed interaction effects between ASC, caspase-1, IL-18, IL-1β and BMI which worsened symptom burden, quality of life, and physical function at both timepoints. Higher acute IL-1β in peripheral blood appears to be a particularly robust predictor of worse mTBI outcomes. Obese BMI is associated with higher acute inflammatory responses to mTBI and may play a role in worse short- and long-term clinical outcomes. Future studies should validate the findings from this preliminary study in a larger, multi-center cohort, evaluate whether the NLRP3 inflammasome is pre-activated in the obese population, and quantify the association of NLRP3 inflammasome proteins with body fat following mTBI.

## Data Availability

Data are available by reasonable electronic request from the principal investigator.
